# Additive Manufacturing and Influencing Factors of Lattice Structures: A Review

**DOI:** 10.3390/ma18071397

**Published:** 2025-03-21

**Authors:** Jinlin Yang, Hui Liu, Gaoshen Cai, Haozhe Jin

**Affiliations:** 1 School of Mechanical Engineering, Zhejiang Sci-Tech University, Hangzhou 310018, China; 2 Key Laboratory of Advanced Manufacturing Technology of Zhejiang Province, School of Mechanical Engineering, Zhejiang University, Hangzhou 310027, China; 3 Institute of Flow-Induced Corrosion, Zhejiang Sci-Tech University, Hangzhou 310018, China

**Keywords:** lattice structure, additive manufacturing, advanced manufacturing technology, topology optimization

## Abstract

Lattice structures have the characteristics of light weight, excellent heat dissipation and mechanical properties. Because of excellent properties, lattice structures have been widely used in aerospace, automobile manufacturing, biomedical and other fields. At present, additive manufacturing is the mainstream method for manufacturing lattice structures. This study reviews the existing literature on additive manufacturing of lattice structures, introduces manufacturing methods, and summarizes the influencing factors of forming quality. In addition, the topology optimization of the unit cell and the gradient design of the lattice structure are discussed, and the future research direction of the lattice structure is proposed.

## 1. Introduction

**Figure 1:**
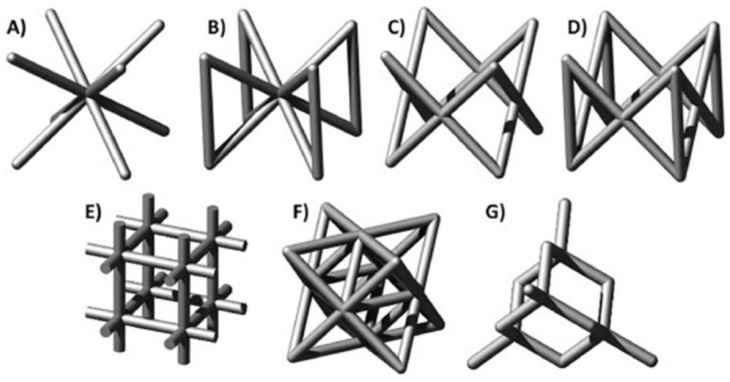
Strut-based lattice structures: (**A**) BCC; (**B**) BCCZ; (**C**) FCC; (**D**) FCCZ; (**E**) cubic; (**F**) octet-truss; (**G**) diamond [2].

**Figure 2:**
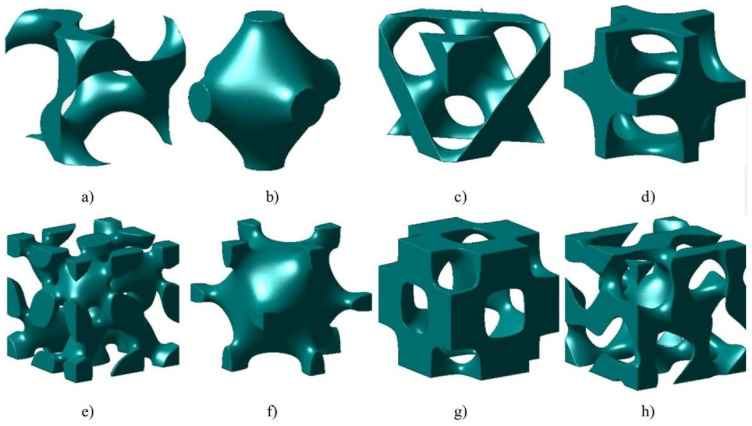
Triply periodic minimal surface unit cells: (**a**) gyroid; (**b**) primitive; (**c**) diamond; (**d**) IWP; (**e**) lidinoid; (**f**) neovius; (**g**) octo; (**h**) split P [3].

**Figure 3:**
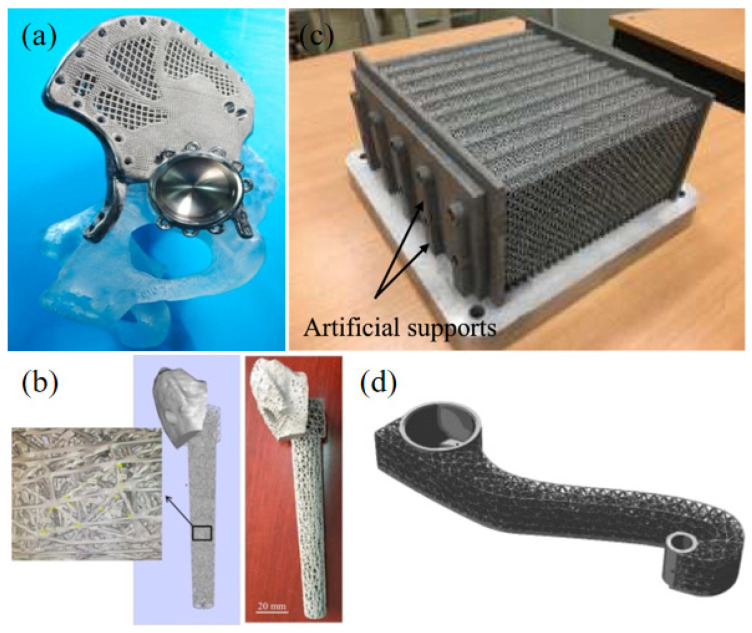
Application of lattice structure: (**a**) hemi-pelvic; (**b**) ankle lattice implant [13]; (**c**) lattice heat exchanger [14]; (**d**) parts for commercial airliners [15].

The lattice structure is a porous structure formed by arranging units, which reduces the weight, realizes lightweight and has high structural strength. The properties of the lattice structure depend directly on the shape and structure of the unit cell [1]. At present, the lattice structures studied more can be divided into two categories. One is the strut lattice structure shown in Figure 1, such as cubic structure, body-centered cubic (BCC) structure, face-centered cubic (FCC) structure and so on [2]. The other is the lattice structure based on the triply periodic minimal surface (TPMS), as shown in Figure 2, namely the TPMS lattice structure [3]. Compared with other solid structures, it has a series of ideal properties such as light weight, noise suppression, high specific strength and stiffness, and good heat dissipation performance [4,5]. It has been widely used in aerospace, automobile manufacturing, biomedical and other fields [1,4,6,7,8,9,10,11,12]. Figure 3 lists some application examples of the lattice structure [13,14,15].

The classical manufacturing techniques of lattice structure mainly include investment casting [16], snap fit [17], expanded metal sheet [18], metallic wire assembly [19], extrusion and electro discharge machining [20], etc. Conventional methods are time-consuming, cumbersome, low material utilization, and difficult to fabricate more complex structures [4]. The emergence and development of additive manufacturing has overcome the limitations of conventional methods. Additive manufacturing requires only a few steps to complete the manufacturing, which greatly saves materials and time costs. Due to the forming characteristics of additive manufacturing, the manufacturing of lattice structures with complex shapes can be realized [21]. Additive manufacturing has become the mainstream method of lattice structures. Although additive manufacturing technology has great advantages, it is largely affected by processing parameters. Improper parameter settings can result in defective parts, for example, high surface roughness, high porosity and high residual stress, etc. [22,23]. At present, many relevant studies have proved that defects can be reduced by adjusting appropriate process parameters and post-treatment [24,25,26,27,28], which will be explained in the following chapters.

In recent years, there have been more and more studies on the density distribution inside the lattice structure, mainly focusing on the graded lattice structure and topology optimization. The reason is that the relative density distribution strategy has a great influence on the mechanical properties. In addition, additive manufacturing improves the freedom of structural design [29].

The article by Junio et al. [30] focuses on the characteristics, production and application of auxetic structures. Joseph et al. [31] focus on assessing the progress, impact and capabilities of additive manufacturing techniques for modern application of lattice structures manufacturing. Isaac et al. [32] paid more attention to the fabrication and structural properties of energy-absorbing structures. Zhang et al. [33] reviewed the fabrication and characteristics of metal lattice structures. Askari [34], Fan [35] and Mazur [36] have introduced the structural characteristics and applications of metamaterials, but there are no reviews of manufacturing methods. The content of the existing review is relatively scattered, and the purpose of this review is to provide as comprehensive a presentation as possible from all aspects of the fabrication and performance factors of lattice structures. In Section 2, the additive manufacturing of lattice structures is summarized. In Section 3, the influence of process parameters on the forming quality of the lattice structure is explained. Section 4 shows that post-processing can be used to optimize the quality of lattice structures. Section 5 provides a discussion from the aspects of structural optimization. Finally, the problems existing in the current lattice structure are put forward, and the future development direction is proposed.

## 2. Additive Manufacturing Processes of Lattice Structures

The development of AM began in the 1980s. Different from conventional methods, the AM process has better adaptability and accuracy, and can use raw materials more effectively, reduce costs and improve material utilization [37]. AM can be divided into several types according to the different materials and energy sources. Some processes use the thermal energy of a laser or electron beam to optically guide the melting or sintering of metal or plastic powders. Other processes spray binders or solvents onto powdered ceramics or polymers through a print head [38,39]. With the maturity of AM, some of these methods have been gradually used to produce lattice structures [40,41,42,43].

### 2.1. Fused Deposition Modeling

**Figure 4:**
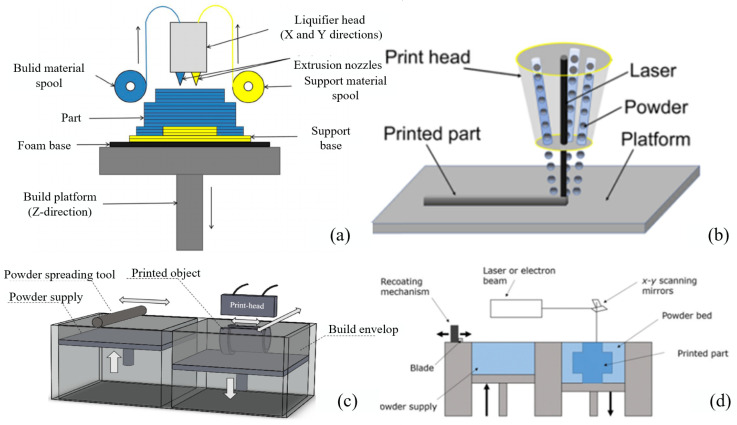
Additive manufacturing method: (**a**) fused deposition modeling [36]; (**b**) directed energy deposition [33]; (**c**) binder jetting [47]; (**d**) powder bed fusion [48].

FDM is a method commonly used to fabricate a lattice structure. FDM has a low maintenance cost and simple printing process. FDM puts the material into a temperature-adjustable nozzle, heats it to melt, squeezes the thermoplastic filament through the nozzle, accurately squeezes and controls the material to stack layer by layer according to the specified path, and the material solidifies to form the final part [44,45]. At the beginning of the FDM process, the material is extruded according to the designed path. The material interacts with the previous deposited material. If the cooling is too fast, there may be voids between the layers, which will significantly reduce the mechanical properties of the produced parts [46]. The principle of FDM is shown in Figure 4a.

**Figure 5:**
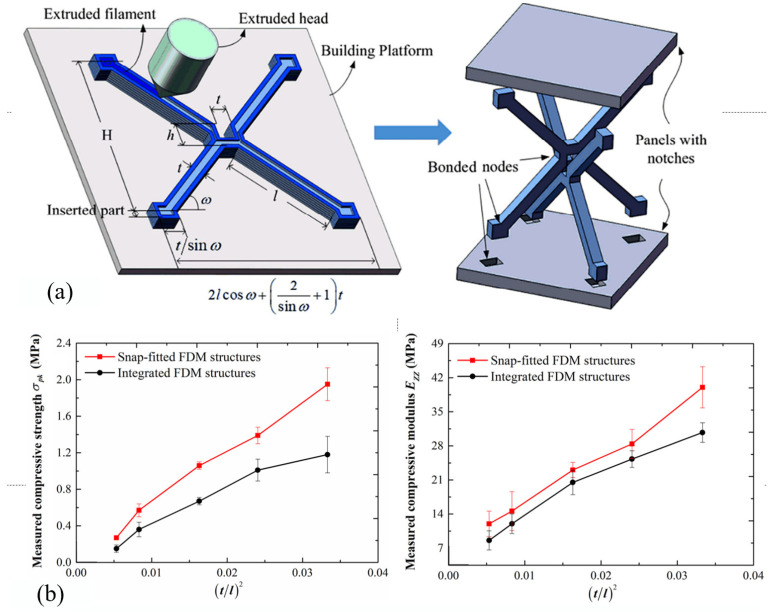
(**a**) Snap fit FDM method; (**b**) comparison of the mechanical properties of integrated FDM structures and snap fit FDM structures [50].

FDM materials are mainly plastic, ceramic and composite materials. The commonly used materials are ABS, PLA and nylon [49]. Alberto et al. [50] used ABS to print the lattice structure and established a lattice damage model. The deformation and damage of lattice structures under compressive loads were observed at the microscopic scale. Kuma et al. [44], inspired by the morphology of sea urchins, used TPU as a material to manufacture a new lattice structure without adding support in the manufacturing process. It can improve the overall manufacturing speed, save materials without affecting quality, and has excellent mechanical properties. Liu et al. [50] proposed a new method to fabricate the lattice structure by combining the FDM with the snap fit method, as shown in Figure 5a. Compared with the conventional FDM process, this method can make all the filaments deposit along the length direction of the pillar, and the fabricated lattice part has good surface finish and low surface roughness. According to the test results of Figure 5b, it can be seen that the mechanical properties have also been significantly improved.

The existing research results prove that this method of combining additive manufacturing with conventional methods is feasible, but the current research is still relatively small, which can become a new direction for future lattice structure manufacturing research.

### 2.2. Directed Energy Deposition

DED is commonly used in metal fabrication, where the raw material can be in the form of powder or wire, and uses lasers or electron beams as energy sources. During manufacturing, the focus is on the surface of the substrate or final sediment layer to form a molten pool. At the same time, the raw material is sent to the molten pool in the form of powder or metal wire. When the heat source moves forward, the deposited metal solidifies on the substrate, and can form arbitrary shapes on both uniform and non-uniform parts by depositing metal materials line by line [33,51,52]. Because of this feature, DED can be used to repair parts, and the low complexity of large component maintenance can use DED [53,54,55]. The principle of DED is shown in Figure 4b.

It has also been used in the fabrication of lattice structures in recent years. Baranowski et al. [56] designed and fabricated two honeycomb topologies with Ti6Al4V alloy powder using laser-engineered net shaping (LENS) in DED, and compared their mechanical properties. Krishna et al. [57] fabricated the CP Ti lattice part by LENS, and found that the porosity and mechanical properties of the adjusted structure can be obtained by changing the process parameters. Dudka et al. [58] fabricated four different sizes of thin-walled honeycomb structures using LENS, and studied the energy absorption property under static and dynamic loading conditions. It was also found that post-treatment is necessary to improve the mechanical properties.

Since DED uses an in situ powder feeding process, the material composition can be flexibly controlled. Therefore, DED is suitable for the manufacture of functionally graded materials. At present, many studies have proved that the graded lattice has better performance than the uniform lattice [59,60,61]. The graded lattice structure will be introduced in Section 5.

### 2.3. Binder Jetting

Binder jetting (BJ) was invented by MIT in 1993 [47]. Binder jetting can work with many material systems, including polymers, ceramics, metals and their composites, and has thus received extensive attention from the scientific and industrial communities. In the printing process, the BJ first selectively sprays the liquid binder onto the powder bed according to the CAD model. The sprayed binder interacts with the powder particles to print the cambium, which is cured by a heater after completion. Then, a new powder is applied over the print layer and the process repeated. This process is repeated until it is completed [62,63,64,65,66,67]. Figure 4c is a typical binder jetting system.

Xu et al. [68] prepared the tensile parts and lattice structure with BJ technology, conducted tensile and compression experiments, and studied the mechanical properties. Xie et al. [69] prepared the TPMS lattice structure via BJ technology, and studied the deformation behavior and compression properties of the structure, finding good plastic deformation capacity and energy absorption capacity. The results showed that BJ can successfully fabricate porous titanium structures with customized porosity changes.

Compared with other AM methods, the time required for BJ to construct parts is short, and the materials available are more extensive. BJ uses a binder instead of an electron beam and laser, which greatly reduces the cost. However, even when there is a great adhesion between the powder and the binder, the strength of the part is still very low. Therefore, post-processing is needed to improve the performance of parts in most cases when BJ is used to manufacture lattice structures [33].

### 2.4. Powder Bed Fusion

PBF is a common method to fabricate lattice structures. Compared with the conventional manufacturing method, PBF can produce lattice materials with higher complexity [70]. PBF uses concentrated thermal energy to selectively melt the area of the powder bed. The main energy sources are laser or electron beams [71,72]. The main process of PBF is as follows: first, the powder is uniformly dispersed in the working area. Then, the laser beam or electron beam is selectively irradiated and melted according to the planned path. Then, the part layer is reduced, and a new layer of powder is laid. The process of layer-by-layer powdering, melting and curing is repeated until the part is manufactured [73,74]. The principle of PBF is shown in Figure 4d.

PBF includes selective laser sintering (SLS), selective laser melting (SLM) and electron beam melting (EBM). The SLS method sinters each atomized particle of the powder together at each edge. The powder particles sintered by SLS are unevenly distributed and the interior is prone to errors. In contrast, the SLM method melts the powder into the surrounding powder, which makes the part layer formed by the melted powder more uniform [75]. Moreover, the parts have a shorter processing time and higher reliability, but heat treatment is recommended to enhance its mechanical properties [76]. In this method, the particle size in the range of 10–150 μm is preferred. The ideal laser energy density is determined by the melting point of the binder or powder, which can be set by adjusting the laser power and scanning speed [77]. The materials used in SLM are mainly alloys in the form of powder. At present, the known materials that can be reliably processed by SLM are still limited. The focus is on aluminum alloys, nickel alloys, steel and titanium alloys, as well as some cobalt alloys, copper alloys, magnesium alloys and pure metals of concern [78]. A large amount of heat is generated during SLM formation. In order to prevent the metal from oxidizing in a high-temperature environment, the building chamber is usually filled with nitrogen or argon to make the forming process in an inert gas [79].

EBM is similar to SLM, but EBM uses electron beam as a heat source [79]. Compared with the laser beam, the electron beam has a higher energy density and efficiency, so the EBM is more efficient. Unlike SLM, EBM needs to be carried out in a highly vacuum environment because the electron beam will collide with the gas molecules, resulting in direction change and energy loss. And manufacturing under high vacuum conditions means a cleaner environment, so EBM has advantages in dealing with active substances [71,80,81].

Ma et al. [82] studied the effect of geometric defects on the mechanical properties of lattice structures prepared by SLM technology and discussed them in detail. Peng et al. [83] used the SLM method to prepare seven groups of lattice structures with different pillar sizes. The effect of the size of the pillar on the mechanical properties of the lattice structure is studied by means of simulation and experiment. Wally et al. [84] verified that SLM can fabricate a series of well-controlled porous Ti6Al4V structures with uniform and graded porosity, and pointed out that SLM as a manufacturing method for dental implants is very promising and more cost-effective than the currently available methods. Jabarzadeh et al. [85] prepared functional fractionated pore samples with SLM, and studied the significant influence of different pore distributions on fracture and deformation behavior. Condruz et al. [86] prepared a variety of lattice structures with SLM using Ti-6Al-4V and Inconel 625 as materials, and evaluated their bending properties.

Table 1 summarizes and compares each additive manufacturing technology. Different technologies have their own advantages and disadvantages. Although these techniques have advantages in building lattice structures, they still face many problems. At present, there is no method for the mass production of lattice structures with a unit length of 10 cm or larger [87]. In addition, the additive manufacturing process is prone to warpage or deformation when manufacturing overhanging structures, and it is necessary to add support to ensure structural stability. However, adding support will increase the time cost and need to minimize the support. A lattice structure also needs to face these problems. These problems in production need further research to achieve large-scale production and improve production efficiency.

## 3. Processing Parameters

Although AM technology can be used to manufacture lattice structures, the quality of lattice structures manufactured by even the same method will be very different. In the manufacturing process, inappropriate parameters may lead to defects, which will adversely affect the quality and mechanical properties of the parts [33,74,88]. Therefore, in order to improve the quality of parts, it is necessary to study the machining parameters [46,88,89].

The processing parameters of FDM include layer thickness, printing temperature, printing speed, etc. Tian et al. [90] used the FDM to fabricate continuous fiber and PLA material samples. It was found that changing the processing parameters can easily control the fiber content of the printed sample, thus affecting the parts. Tang et al. [91] fabricated the lattice structure of PLA by FDM, and analyzed the effect of processing parameters on the sample. With the increase in printing temperature, the tensile strength and elastic modulus increased first and then decreased, and the mechanical property of the lattice structure decreased. As the printing speed increased, the tensile strength and elastic modulus also increased. Wu et al. [92] studied the effects of layer thickness and raster angle on the mechanical properties of 3d printed PEEK. The results showed that the raster angle and layer thickness have a significant effect on the tensile, compressive and three-point bending properties of the material.

**Figure 6:**
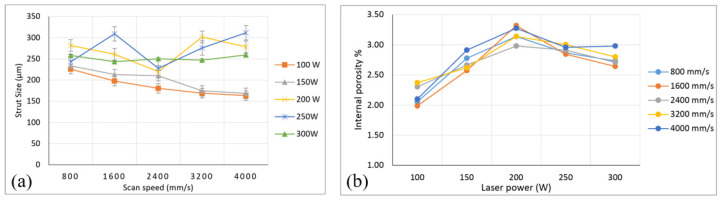
Influence of laser power and scan speed: (**a**) strut size; (**b**) influence of laser power internal porosity [22].

The processing parameters of SLM mainly include scanning speed, scanning spacing, laser power, layer thickness and layering mode [25,93]. Salem et al. [22] fabricated the Ti6Al4V lattice structure by SLM to study the influence of laser power and scanning speed. The effects of these two parameters on the size and internal porosity of the strut were studied by scanning electron microscopy (SEM) and optical microscopy (OM). The results are shown in Figure 6. The strut size decreases with increasing scan speeds for low-laser-power conditions, while at the highest laser powers, insignificant differences were observed at various scan speeds. Sing et al. [94] fabricated a new type of titanium tantalum alloy sample by SLM. The SLM processing parameters have a significant effect on the dimensional accuracy, porosity, yield strength and elastic modulus. The mechanical properties are affected by laser correlation and layer thickness. More than any other factor, the support size of the lattice structure is most sensitive to laser power. Controlling the processing parameters can not only improve the dimensional accuracy of the lattice structure, but also better control the mechanical properties.

In addition, Sairam et al. [95] fabricated lattice structures with different shapes by B, and studied the effects of sintering time, sintering temperature and other process parameters on these parts. The results show that the sintering time and temperature will change the sintering mechanics and affect the performance of the lattice.

Scanning strategy is also an important factor. Scanning strategy is the motion behavior of energy beam such as laser and electron beam. The scanning strategy has a certain effect on the microstructure and mechanical properties of the structure. Many researchers have studied the scanning strategy to improve the quality of parts [96,97].

Radek et al. [97] studied the lattice structure of the AlSi10Mg powder material fabricated by SLM, and the production of laser strategies and parameters recommended by suppliers would lead to defects. They changed the default bending scanning strategy of AlSi10Mg powder material to the contour strategy. The results showed that the contour strategy reduces the porosity of the part and improves the accuracy.

**Figure 7:**
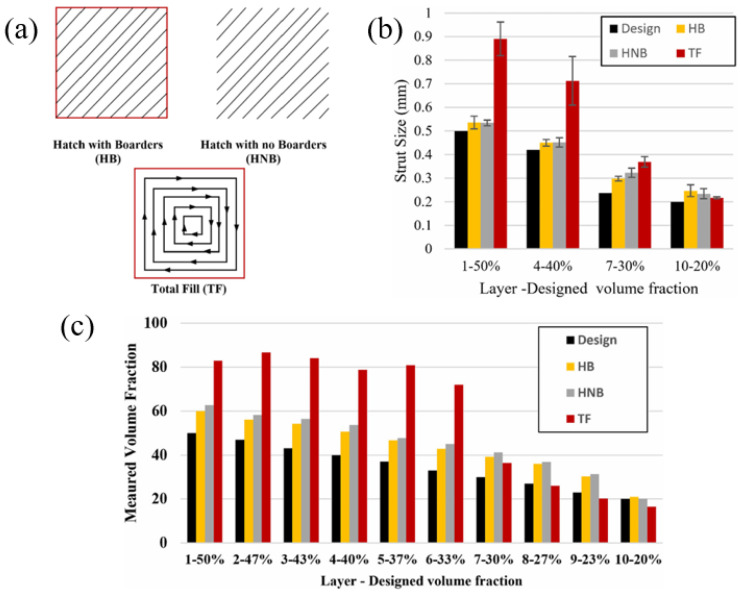
(**a**) Different scanning strategies; (**b**) strut size compared to design values; (**c**) volume fraction compared to design values [98].

Mahmoud et al. [98] proposed three different scanning strategies to make pore gradient lattice structures, as shown in Figure 7a, and studied their effects on volume fraction and support size. It can be seen from Figure 7b,c that the scanning strategy has a significant effect. The deviation values of volume fraction and pillar size in each layer of the parts manufactured by different scanning strategies are different. The above research results show that the scanning strategy has a real effect on the quality of the parts.

The simulation of the AM process will be a powerful tool to optimize process parameters and improve forming quality [99]. By simulating the forming of different materials in different parameters, it can save the cost and improve the quality of future parts manufacturing. More research is necessary to obtain more reasonable parameters for manufacturing lattice structures.

## 4. Post-Processing

The process limitations of additive manufacturing or the selection of process parameters will more or less lead to defects in the lattice structure, such as high surface porosity and high surface roughness, which will affect the mechanical properties and the manufacturing of parts. Faced with this problem, the researchers chose to improve the reliability of the product through post-processing adjustments or performance enhancements. However, at present, there are few relevant studies in this area, so the post-processing of complex lattice is still challenging and needs further research. In this paper, the existing post-treatment of lattice structures is reviewed. The common post-treatment methods include surface modification, heat treatment and hot isostatic pressing.

### 4.1. Surface Modification

**Figure 8:**
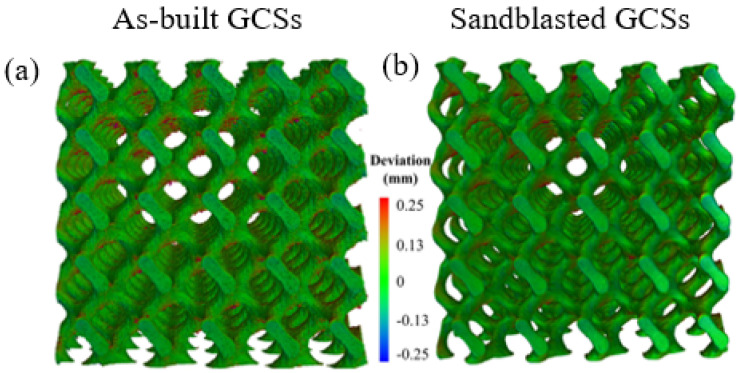
Surface of lattice structure before and after post-treatment [100].

The lattice structure manufactured by AM may have poor surface quality, and surface modification is needed to improve the surface quality. Surface modification methods include finishing, shot peening, sandblasting, electrochemical polishing, chemical etching, etc. Yang et al. [100] found that sandblasting post-treatment can remove the bonded powder particles during the powder bed fusion process, making the surface smooth. The sandblasted surface is shown in Figure 8, which improves the fatigue resistance of gyroid cellular structures (a typical TPMS structure). Chemical etching and electrochemical polishing are also commonly used techniques. Pyka et al. [101] used the electrochemical polishing method to reduce the surface roughness of Ti6Al4 V porous structure, which greatly reduced the roughness difference between the top and bottom of the supporting surface, making the whole structure obtain more controllable and uniform roughness, but the mechanical properties of the structure decreased after treatment. Hooreweder et al. [102] used a mixture of hydrochloric acid and hydrogen peroxide to treat the surface of the cobalt–chromium F75 bracket produced by SLM by chemical etching, which not only removed the particles attached to the surface, but also improved the surface finish and maintained the quasi-static and fatigue properties of the parts.

### 4.2. Heat Treatment

The microstructure of AM parts has an obvious influence on its mechanical properties [23]. Heat treatment is usually used to reduce the thermal stress inside the part and control the microstructure of the material. Li et al. [103] studied the effect of heat treatment on AlSi10Mg samples fabricated by SLM. It had been found that the microstructure and mechanical properties of the alloy can be controlled by different heat treatments. Maskery et al. [104] proved that the deformation behavior of the SLM aluminum lattice structure can be improved by heat treatment. The heat-treated part absorbed the same energy as the built lattice structure under compression deformation, but they experienced significantly lower peak stress. Liu et al. [105] prepared the lattice structure of Ti-24Nb-4Zr-8Sn and observed the mixed microstructure of the α + β phase. After heat treatment, only β phase existed in the specimen tissue, the compressive strength increased and Young’s modulus decreased. The effect of heat treatment on reducing the internal residual stress of production parts and the structure of customized production parts was verified.

### 4.3. Hot Isostatic Pressing

Hot isostatic pressing (HIP) can reduce the porosity and improve the density of parts, but it is difficult to play a role in surface cracks and pores. Tillmann et al. [106] studied the effect of HIP processing parameters on the density and microstructure of IN718 SLM parts. It was found that HIP can densify most of the pores, but it can’t achieve 100% density through HIP due to the capture of inert gas in SLM. Mower et al. [107] carried out hot isostatic pressing post-treatment on DMLS Ti6Al4V and SS316L, which significantly improved the fatigue strength. Wu et al. [108] designed SLM Ti-6Al-4V lattice structure and studied the effect of HIP on its fatigue properties. It was found that HIP treatment at a high temperature of 1000 °C/150 MPa can eliminate the pores in the strut. The fatigue durability ratio of the lattice increased from 0.3 to 0.55, and the fatigue performance was improved. Wu et al. [109] also conducted another study to observe the effect of HIP on the microstructure, densification, flexural strength, impact toughness and fracture behavior of Ti-6Al-4V alloy. Due to the elimination of lamellar pores and low-toughness α′-martensite, the impact energy of vertical and horizontal alloys increased by 560% and 190%, respectively. The anisotropy in bending and impact properties had also been fully alleviated.

Although it has been confirmed that post-processing can improve the quality of lattice structure, the current research is still very limited, and post-processing may cause some damage while improving the quality of parts. For example, electrochemical polishing is an ideal method to improve surface finish, but too-long contact time with a chemical solution will weaken the quality of parts and reduce mechanical properties. In the face of such problems, it is necessary to find or improve post-processing methods to reduce the damage to parts, which is a work worth exploring.

## 5. Structural Optimization and Design

### 5.1. Design of the Lattice Structure

The cell is the most basic element that makes up the entire lattice structure. It can design lattice by performing Boolean operations on geometric figures, which is usually used for a lattice of truss class. There are also methods based on implicit surface through the establishment of mathematical equations to achieve the establishment of the structure, such as a TPMS lattice that is generated by this method. And topology optimization methods can also be used to create entirely new units. Additionally, 3-Matic (Materialise, Leuven, Belgium), Within (Autodesk, San Francisco, CA, USA), Optistruct (Altair, Troy, MI, USA) and Rhino (McNeel, Barcelona, Spain) are all available as design tools [110]. Comsol, ANSYS, and ABAQUS are all conventional software that can be used to perform the finite element analysis of lattice structure performance to verify the rationality and reliability of the design.

### 5.2. Topology Structure

The unit cell and arrangement of the lattice structure have a great influence on the overall performance. Topology optimization is a design method that maximizes structural performance by optimizing material distribution, automatically seeking the best material layout under given constraints. Widely used in aerospace, mechanical engineering and other fields, can significantly reduce weight and improve performance [111]. Topologically optimized parts usually have complex shapes and are difficult to fabricate by ordinary methods The emergence of additive manufacturing technology makes the fabrication of topological structures convenient and promotes the related research.

**Figure 9:**
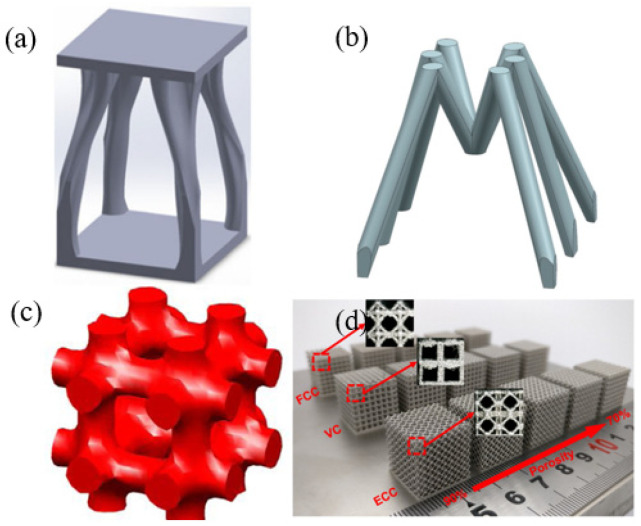
(**a**) Bionic cuttlefish bone [113]; (**b**) ant [114]. (**c**) Topological structure designed by Zhang et al. [115]: (**d**) FCC, VC, ECC [116].

**Figure 10:**
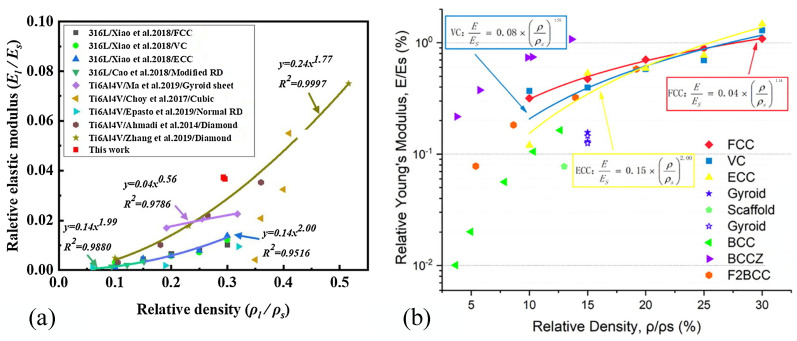
(**a**) Comparison between the topology optimization structure and relative elastic modulus of the structure [115]. (**b**) Comparison of properties of different lattice structures [116].

Cuttlebone is a natural material with desirable mechanical properties such as high compressive strength, high porosity and high permeability. These properties are sought after in both biomimetic and biomedical structural materials. Inspired by the microstructure of cuttlefish bone [112]. Hu et al. [113] designed the lightweight multi-functional lattice structure, as shown in Figure 9a. Li et al. [114] observed and analyzed ants in a weight-bearing state, where the position of their legs kept them stable. Inspired by this, the lattice structure is designed to support the payload of Fengyun-3 satellite, as shown in Figure 9b, ensuring the stiffness and strength while achieving the lightweight design goal. Zhang et al. [115] used SLM to fabricate the topologically optimized cell, as shown in Figure 9c. The finite element analysis was performed using COMSOL software, and its mechanical properties were studied experimentally. The lattice structure is compared with that in other studies, as shown in Figure 10a. The results show that the relative elastic modulus of the designed lattice structure is better than most of the current lattice structures. Xiao et al. [116] combined AM and topology optimization to design three kinds of structures: center cube (FCC), vertex cube (VC) and edge-center cube (ECC), as shown in Figure 9d, and evaluated their mechanical properties. Figure 10b shows the comparison of these three structures with various lattice structures made of 316 L stainless steel, which proves that the performance of the topologically optimized is better than that of most lattice structures.

Moreover, the design of topology optimization that combines a lattice structure with solid has also been proposed, and it has great performance. Wang et al. [117] proposed a multi-scale design method, combining topology optimization and lattice-based optimization to create a solid–lattice hybrid structure in order to improve the mechanical properties and reduce weight. Two typical aerospace structures were optimized and the superiority of the structures was verified by experiments. The results showed that the solid–lattice hybrid design significantly improves the stiffness and natural frequency compared with the pure solid design and the pure lattice design. Dong et al. [118] proposed a design method of a solid–lattice hybrid structure, and used this method to design a three-point bending beam. The superiority of the design was verified by a simulation and experiment. The simulation results showed that the mechanical properties of the solid–lattice hybrid structure are the best. The experimental results showed that the stiffness and ultimate strength of the hybrid structure are better than those of the pure lattice and pure solid structure.

A reasonable topology has better performance than a general structure, but there is still a lot of research space for the design of new topological shapes. New unit shapes need to be designed to meet the requirements of lightweight and versatility. A bionic structure has important reference significance. In addition, the lattice–solid hybrid structure also has excellent performance, but there are few related studies and great development potential.

### 5.3. Graded Structure

With the research and development of lattice structure, gradient lattice has been proposed. Compared with the uniform lattice structure, the cells of the gradient lattice structure can show different relative densities in any direction. The design of a graded lattice structure is usually to change the strut diameter, unit type, material composition and unit size [1]. At present, the research on a graded lattice structure focuses on the influence of various graded strategies on the mechanical properties.

**Figure 11:**
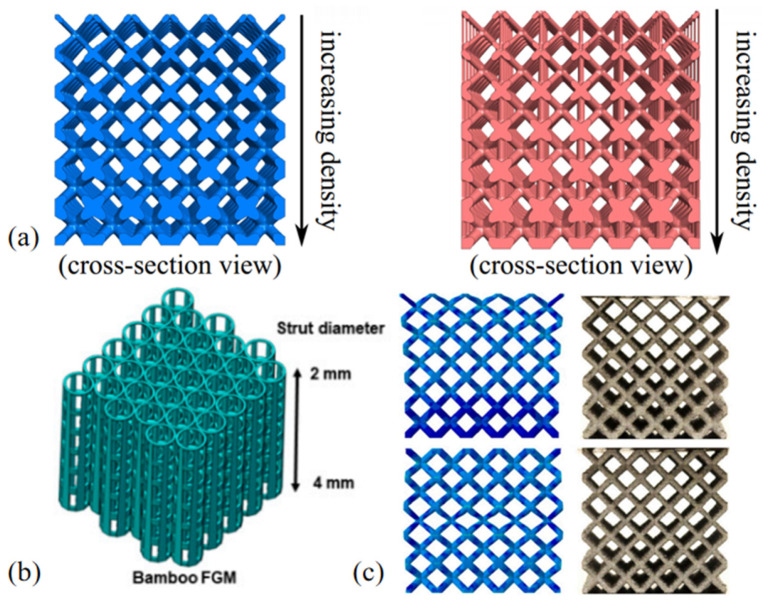
(**a**) BCC and BCCz graded structures [119]; (**b**) bionic bamboo lattice structure [120]; (**c**) unidirectional and bidirectional graded lattice structures [121].

**Figure 12:**
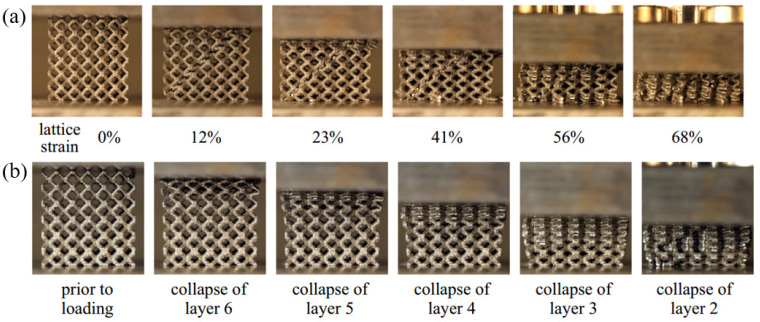
The deformation modes of different lattice structures: (**a**) uniform lattice structure; (**b**) graded lattice structure [59].

Maskery et al. [119] studied the BCC and the reinforced variant BCCz, and fabricated their uniform and graded structures, as shown in Figure 11a. Quasi-static compression experiments were carried out to compare the performance; the results show that the energy absorption of the gradient structure is 114% higher than that of the uniform structure, at 1371 ± 9 kJ/m^3^ and 640 ± 10 kJ/m^3^, respectively. In their other paper, the compressive deformation behavior of uniform and graded lattice structures is introduced. As shown in Figure 12, the uniform structure will produce lateral expansion, vertical crack propagation, and eventually form a 45° shear failure, and the graded lattice structure exhibits a layer-by-layer failure deformation mode [45]. Inspired by bamboo, Wen et al. [120] created a bionic bamboo lattice structure with a gradual change in strut diameter, as shown in Figure 11b. Through quasi-static compression experiments, it was found that the main failure mode is the breakage of the laminated layer, starting from the support layer with a low relative density to the part with a high relative density, while the initial collapse of the uniform lattice structure occurs randomly. Based on BCC, Rodrigo et al. [121] designed graded lattice structures with unidirectional and bidirectional density grading, as shown in Figure 11c. The plateau stress and energy absorption capacity of the bidirectional density graded lattice are higher than those of the uniform and unidirectional density graded lattice structure. The finite element simulation shows that the enhancement of strength and energy absorption is related to the periodic collapse of the graded structure corresponding to the density graded strategy. Lin et al. [122] proposed five kinds of graded functional lattice structures with different densities perpendicular to the loading direction. Through finite element simulation and experiments, it was found that when the loading direction is perpendicular to the density graded forming direction, the failure mode of the graded lattice structure is almost the same as that of the uniform structure, showing a certain angle shear failure. The high-density part of the lattice structure is the main bearing part, and the greater the density difference between the two ends of the lattice structure, the greater the elastic modulus. The maximum elastic modulus, yield strength and compressive strength of gradient structure are 28.99%, 16.77% and 14.46% higher than those of uniform structure, respectively.

Compared with uniform lattice structures, graded lattice structures usually have different deformation behaviors and better mechanical properties. The density distribution has a great influence on the mechanical properties of graded lattice structures. Exploring more density distribution forms can promote the development of graded lattice structures. The existing research adopts the most variable strut diameter graded strategy. The graded strategy has a continuous distribution of relative density, which is easy to control, and the structural graded design is simple and feasible. There is a lack of research on complex graded change mode, and there is a problem of node connection in changing the graded change mode of element structure size and structure type. The transition at the node is abrupt, which makes it is easy to produce sharp stress concentration, resulting in the decrease in the overall mechanical properties of the graded lattice structure. If we want to study a more complex graded structure, a way to make the node transition smoother is an important problem to be solved.

## 6. Conclusions

The excellent properties of lattice structures have attracted wide attention, as they have great potential in the fields of biomedicine, automobile and aerospace. In this study, the additive manufacturing methods, quality influencing factors and performance improvement design of lattice structures were sorted out and summarized through literature search.

In the face of the drawbacks of the additive manufacturing process, it is necessary to find the optimization measures of process parameters and appropriate post-processing methods to improve the quality of parts. Simulation of the additive manufacturing process will be a powerful tool to optimize process parameters and improve forming quality.

A reasonable topological design can bring good performance, and a bionic structure and solid–lattice hybrid form can be used as future research objects. In order to meet the requirements of light weight and versatility, it is necessary to design new unit shapes, and bionic structures will be a great design direction.

A graded lattice structure has greater advantages than a uniform lattice structure. The mechanical response and failure mechanism of the graded lattice structure are closely related to the graded mode. The gradient strategies in the existing research are relatively simple and lack the design and research of complex gradient lattice structures, so it is possible to design gradient lattice structures combining multiple gradient strategies. At the same time, the design of graded structures needs to solve the node connection as an important problem.

## Figures and Tables

**Table 1:** Additive manufacturing of lattice structures.

Method	Material	Principle	Characteristic	Refs.
FDM	Metal, thermoplastic materials	The material is melted and extruded in the nozzle, cooled and solidified	Low maintenance cost and simple printing process	[44,45,46,49,50]
DED	Metal, ceramic	The material is melted and cooled to solidify	Flexible control	[51,55,56,57,58]
BJ	Metals, ceramics, polymers	Sprayed and sewed with adhesive	Low cost, wide range of available materials, low part strength	[33,68,69]
PBF	Metal, ceramic, thermoplastic materials	The material is melted and cooled to solidify	High material utilization rate, high precision	[70,75,76]
